# Supplementary figures and images for: The mechanical energetics of walking across the adult lifespan
Source: PLoS One. 2021 Nov 12;16(11):e0259817. doi: 10.1371/journal.pone.0259817 (PMC8589218; doi:10.1371/journal.pone.0259817)

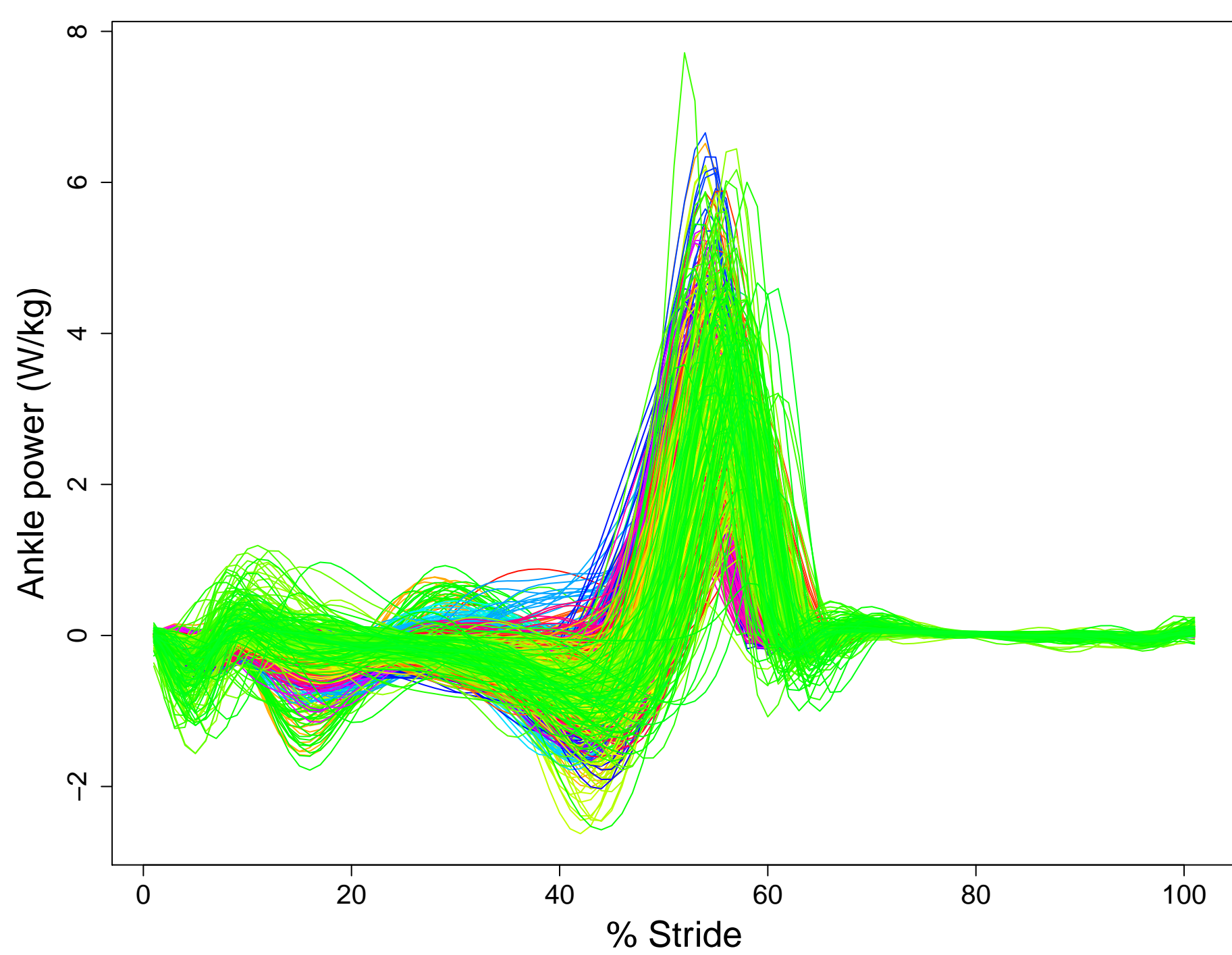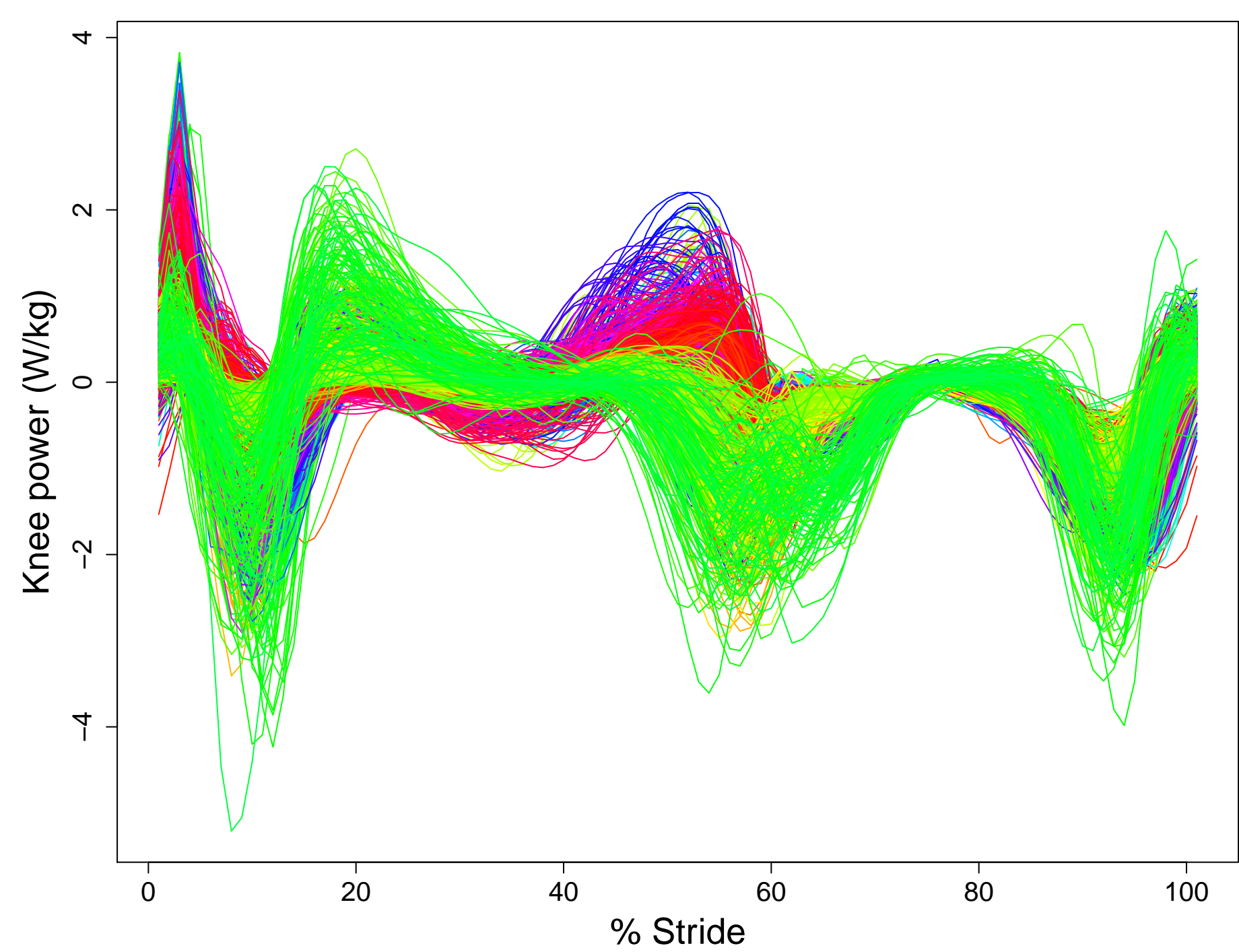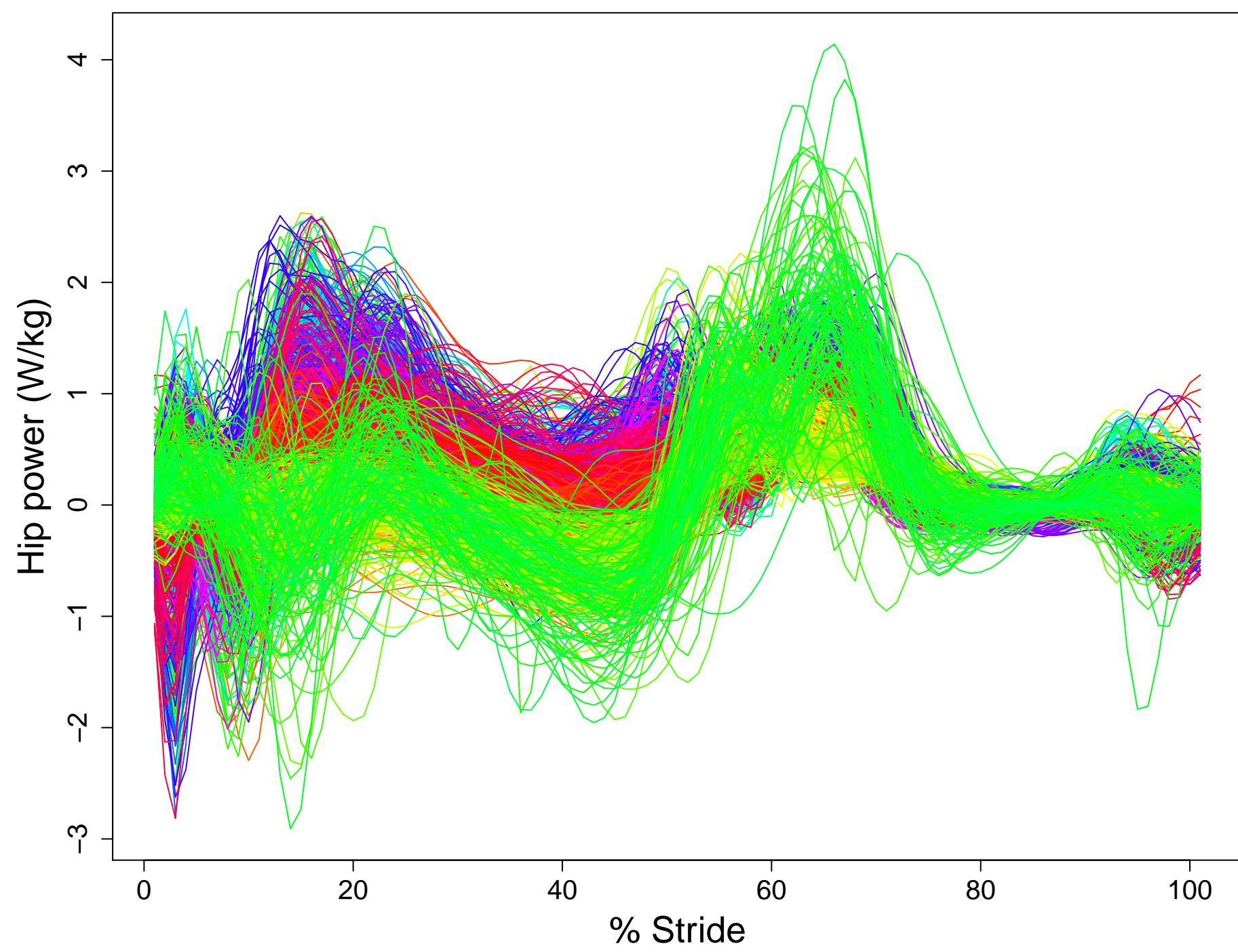

Supplement: S1 Fig — (PDF) [file pone.0259817.s001.pdf]

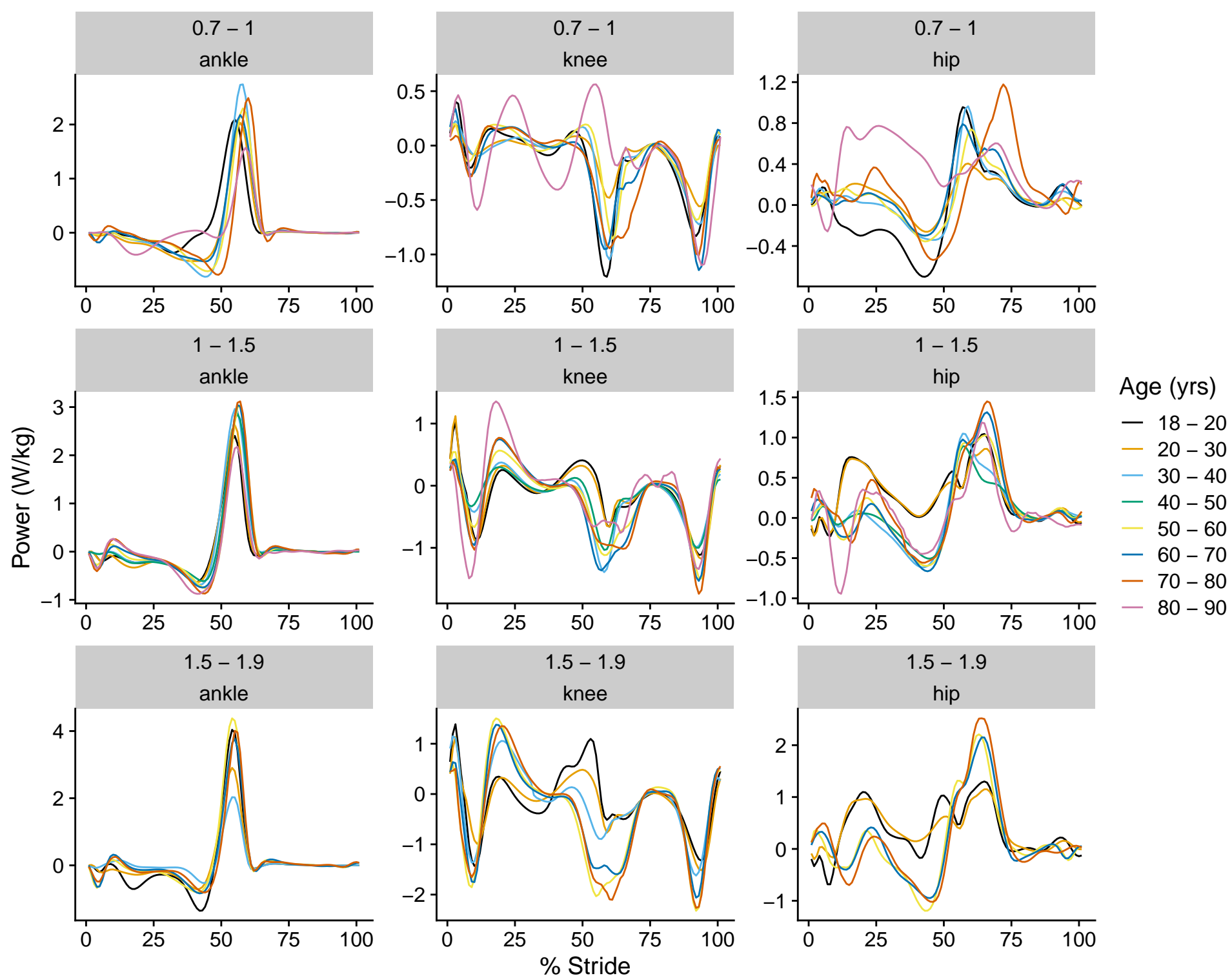

Supplement: S2 Fig — (PDF) [file pone.0259817.s002.pdf]
