# Supplementary material for: The mechanical energetics of walking across the adult lifespan
Source: PLoS One. 2021 Nov 12;16(11):e0259817. doi: 10.1371/journal.pone.0259817 (PMC8589218; doi:10.1371/journal.pone.0259817)
Supplement: S1 Table — (DOCX) [file pone.0259817.s003.docx]

Table SM1. Brief methodologies of included studies

|  | Fukuchi et al. | Horst et al. | Schriber & Moissnenet | Taylor et al. |
| --- | --- | --- | --- | --- |
| Country | Brazil | Germany | Luxembourg | England |
| Inclusion criteria | Healthy, free from lower limb injuries | Physically active, without gait pathology and free of lower extremity injuries | Asymptomatic, i.e. healthy and injury free for both lower and upper extremities | Live independently, be independent walkers, with no surgical procedures |
| Camera system | 12 cameras (Raptor-4, Motion Analysis Corporation, Santa Rosa, CA, USA) | 10 cameras (Oqus 310, Qualisys, Gothenburg, Sweden) | 10 cameras (Oqus 4, Qualisys, Gothenburg, Sweden) | 7 cameras (T20, Vicon, Oxford, UK) |
| Camera sampling frequency | 150Hz | 250Hz | 100Hz | 100Hz |
| Force platform system | Dual-belt, instrumented treadmill (FIT, Bertec, Columbus, OH, USA) | 2 force plates (Type 9287CA, Kistler, Switzerland) | 2 force plates (OR6-5, AMTI, Massachusetts, USA) | 1 force plate (Type 9281CA, Kistler, Winterthur, Switzerland) |
| Force sampling frequency | 300Hz | 1000Hz | 1500Hz | 1000Hz |
| Footwear | Unshod | Unshod | Unshod | Shod |
| Surface | Treadmill | Overground | Overground | Overground |
