# Supplementary material for: The mechanical energetics of walking across the adult lifespan
Source: PLoS One. 2021 Nov 12;16(11):e0259817. doi: 10.1371/journal.pone.0259817 (PMC8589218; doi:10.1371/journal.pone.0259817)
Supplement: S2 Table — (DOCX) [file pone.0259817.s004.docx]

Table SM2. 29 location, scale, shape, distributions used in Bayesian Optimization

| Distribution | Identifier in R software |
| --- | --- |
| Exponential generalized Beta type 2 | EGB2 |
| Exponential Gaussian | exGAUS |
| Generalized t | GT |
| Gumbel | GU |
| Johnson's SU | JSU |
| original Johnson's Su distribution | JSUo |
| Logistic | LO |
| Normal Exponential t | NET |
| Normal | NO |
| Power Exponential | PE |
| Power Exponential type 2 | PE2 |
| Reverse Gumbel | RG |
| Skew Power Exponential type 1 | Sep-01 |
| Skew Power Exponential type 2 | Sep-02 |
| Skew Power Exponential type 3 | Sep-03 |
| Skew Power Exponential type 4 | Sep-04 |
| Shash | SHASH |
| Shash original | SHASHo |
| Shash original type 2 | SHASHo2 |
| Skew Normal Type 1 distribution | SN1 |
| Skew Normal Type 2 distribution | SN2 |
| Reparametrised skew t type 3 | SST |
| Skew t type 1 | ST1 |
| Skew t type 2 | ST2 |
| Skew t type 3 | ST3 |
| Skew t type 4 | ST4 |
| Skew t type 5 | ST5 |
| t-distribution | TF |
| t distribution reparametrised | TF2 |
