# Supplementary material for: The mechanical energetics of walking across the adult lifespan
Source: PLoS One. 2021 Nov 12;16(11):e0259817. doi: 10.1371/journal.pone.0259817 (PMC8589218; doi:10.1371/journal.pone.0259817)
Supplement: Attachment — Submitted filename: ResponseReviewers_R2.docx [file pone.0259817.s006.docx]

# Comments are in bold, response in normal typeset, and *excerpts from manuscript are in italics.*

# Journal Requirements

## Please review your reference list to ensure that it is complete and correct. If you have cited papers that have been retracted, please include the rationale for doing so in the manuscript text, or remove these references and replace them with relevant current references. Any changes to the reference list should be mentioned in the rebuttal letter that accompanies your revised manuscript. If you need to cite a retracted article, indicate the article’s retracted status in the References list and also include a citation and full reference for the retraction notice.

Reply: We are unaware that any of our citations are that of retracted articles. We kindly request the Editorial team to flag such an instance if an error has been made.

# Reviewer #1

## Reviewer #1: First of al, my apologies for my delayed response. Thanks to the authors for replying to my comments regarding the manuscript. I think that some of the replies, particularly those related to the differences (tread vs overground and shod and unshod) between studies and the rationale for putting them together should be part of the introduction or at least should be address in the methods (not only in the limitations). I really appreciate authors present their findings in relation to the contrast with most literature in the field. This study will be certainly controversial, even more considering potential implications for rehabilitation.

Reply: We thank the Reviewer for this comment. We agree that this is a controversial paper, hence we aim to be very transparent in our analysis. We have included the rationale in the rationale in the Methods, which reads as in L97:

Despite the presence of some methodological variations between the presently included studies, data pooling was deemed appropriate to conduct based on several reasons. First, a previous meta-analysis [3] pooled data into a random-effects model despite methodological variations in the primary studies (e.g. barefoot walking [6] and shod walking [22]). The present analysis also adopted a random effects modelling approach. Second, a previous study reported no significant differences in A2 and H3 powers between treadmill and overground walking [23]. Third, given that walking without shoes reduces step length compared to shoes [24], to account for between-study variation in footwear presently, we included step length as a covariate in our models.

We have also included in L76 of the Introduction a sentence on our study design:

To achieve this aim, we pooled together the individual participant data of three publicly available datasets [16-18], and the data from one primary research.

Minor
I think you should put studies by surname with capital letter?

Reply: We thank the Reviewer for this comment. We apologise to the Reviewer because we are uncertain as to the source of the problem. Is the Reviewer referring to any in-text citations, or the formatting of the bibliography? We have tried to follow all formatting to Plos ONE requirements.

Example of our formatting below

Example of a Plos ONE publication (doi: 10.1371/journal.pone.0238690)

*Line 188: typo. “gaitcycle”*

Reply: We have corrected this to read as “gait cycle”.
